# Supplementary material for: Discovery of deep-sea coral symbionts from a novel clade of marine bacteria with severely reduced genomes
Source: Nat Commun. 2024 Nov 4;15:9508. doi: 10.1038/s41467-024-53855-5 (PMC11535214; doi:10.1038/s41467-024-53855-5)
Supplement: Supplementary file 1 — Supplementary Information [file 41467_2024_53855_MOESM1_ESM.pdf]

Supplemental Information for:

Discovery of deep-sea coral symbionts from a novel clade of marine bacteria with severely reduced genomes

Samuel A. Vohsen, Harald R. Gruber-Vodicka, Santiago Herrera,

Nicole Dubilier, Charles R. Fisher, Iliana B. Baums*

*Corresponding Author: Iliana.baums@hifmb.de

Table of Contents

**Supplementary Table S1**	Page 2
**Supplementary Table S2**	Page 3
**Supplementary Table S3**	Page 4
**Supplementary Table S4**	Page 5
**Supplementary Figure S1**	Page 6
**Supplementary Note 1**	Page 7
**Supplementary Figure S2**	Page 8
**Supplementary Figure S3**	Page 9
**Supplementary Figure S4**	Page 10
**Supplementary Figure S5**	Pages 11-12

Table S1: Collection year, sampling site, preservation method, and extraction procedure for coral, water, and sediment samples used in 16S rRNA amplicon sequencing. All samples were flash frozen and extracted with powersoil kits unless otherwise noted. Underlined sample numbers indicate preservation in ethanol. A star indicates sample numbers extracted with allprep kits. Where colonies were sampled multiple times, parentheses indicate the number of colonies represented. Brackets indicate the number of colonies where subsamples were processed with both powersoil and allprep kits. Italics indicate water samples from niskin bottles processed with powerwater kits. A carrot indicates water samples using a McLane pump and the filter was both preserved in ethanol and frozen.

		Site							
Sample Type	Year	GB299	VK862	MC751	VK826	GC234	MC885	GC249	GC290
C. americana	2010	2*[1]	2*	-	-	-	-	-	-
C. delta	2015	-	-	<u>11</u>	-	<u>5</u>	<u>12</u>	-	-
	2016	-	-	6	3	15	17 (14)	-	-
	2017	-	-	19 (9)	-	20 (18)[5]	-	14	10
Sediment	2015	-	-	6	-	1	6	-	-
	2016	-	-	4	2	8	5	-	-
	2017	-	-	5	-	4	-	3	-
Water	2015	-	-	1^	-	1^	-	-	-
	2016	-	-	-	-	*6*	*5*	-	-
	2017	-	-	*6*	-	*3*	*3*	*1*	-

Table S2: Metadata of samples used for metagenomics and microscopy. Numbers indicate the year that the sample was collected that was used for each analysis. Underlining indicates which microscopy samples bacteria were detected in. PE = paired-end sequencing

Site	Colony Name	Metagenomes Deep (150bp PE)	Metagenomes (150bp PE)	Metagenomes (100bp PE)	Meta-transcriptome	FISH	TEM
MC751	E7-2	-	-	17	-	-	-
	E7-4	-	-	16	-	-	-
	E7-6	-	-	15	-	-	-
	E7-8	-	-	16	-	-	-
	S11-1	-	-	17	-	-	<u>17</u>
	S11-3	-	-	17	-	-	<u>17</u>
	S11-9	-	16	16	16	<u>16</u>	-
	S14-2	16	16	-	-	<u>16</u>	-
	S14-3	-	-	17	-	-	-
	S14-4	-	-	17	-	-	-
	S14-5	-	16	16	-	-	-
	S17-1	-	-	17	-	-	<u>17</u>
	S17-3	-	-	15	-	-	-
	S17-4	-	16	16	-	-	-
	T4-4	-	-	17	-	-	<u>17</u>
	T4-6	-	-	17	-	-	<u>17</u>
MC885	S31-1	-	-	16	-	-	-
	S31-2	-	-	16	-	-	-
	S31-3	-	16	16	-	<u>16</u>	-
	S3-14	16	16	-	16	<u>16</u>	-
GC234	S33-16	-	16	-	-	16	-
	S34-11	-	16	-	-	-	17

Table S3: Distances between *Callogorgia* samples. The minimum distances between *Callogorgia* samples from different sites are presented in kilometers at the top of the table. The final row displays the maximum distance between *Callogorgia* samples collected within a site.

	GB299	GC234	GC290	GC249	MC751	MC885	VK862	VK826
GB299	-	108 km	155 km	168 km	244 km	250 km	407 km	443 km
GC234	108 km	-	57 km	70 km	148 km	152 km	308 km	350 km
GC290	155 km	57 km	-	14 km	100 km	100 km	272 km	304 km
GC249	168 km	70 km	14 km	-	88 km	87 km	259 km	291 km
MC751	244 km	148 km	100 km	88 km	-	16 km	172 km	204 km
MC885	250 km	152 km	100 km	87 km	16 km	-	175 km	204 km
VK862	407 km	308 km	272 km	259 km	172 km	175 km	-	37 km
VK826	443 km	350 km	304 km	291 km	204 km	204 km	37 km	-
max within site	0 m	223 m	180 m	32 m	177 m	1514 m	97 m	70 m

Table S4: Primer sequences used for PCR and FISH microscopy. Linkers are underlined.

Primer Name	Sequence (5' – 3')	Use
27F + CS1 linker	<u>ACACTGACGACATGGTTCTACA</u>AGAGTTTGATCMTGGCTCAG	PCR
355R + CS2 linker	<u>TACGGTAGCAGAGACTTGGTCT</u>GCTGCCTCCCGTAGGAGT	PCR
EUB338 I	GCTGCCTCCCGTAGGAGT	FISH
EUB338 II	GCAGCCACCCGTAGGTGT	FISH
EUB338 III	GCTGCCACCCGTAGGTGT	FISH

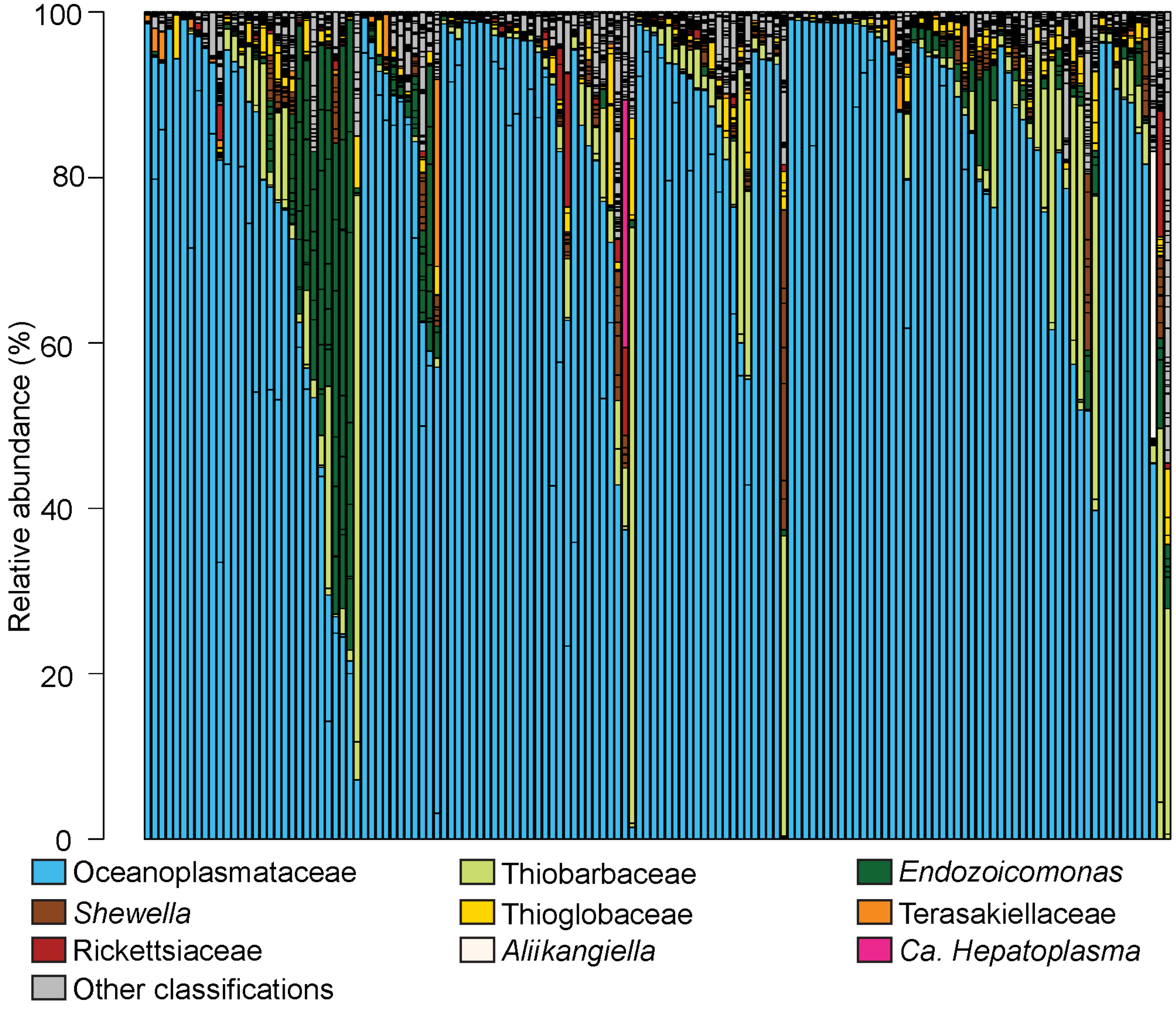

Fig. S1: Other microbes detected in *Callogorgia*

Barplots of all ASVs detected in *Callogorgia* samples based on 16S rRNA amplicon sequencing. ASVs from nine most abundant taxa are represented in colors. ASVs with other classifications are displayed in gray. Each column represents the microbial community of a single sample. Colonies are organized by species, site, and sampling year. *C. delta* sites are ordered left to right by increasing depth.

Supplementary Note 1

To investigate the possibility that sediment samples were contaminated with *Ca. Oceanoplasma callogorgiae* and *Ca. Thalassoplasma callogorgiae,* we screened an additional forty sediment samples from sites lacking *Callogorgia* that were sequenced on the same runs. These samples span a range of depths from 60 to 1800 meters and include four from mesophotic sites. The metadata associated with these samples can be found in Supplementary File 1: Tab A. ASVs Molli-1 and Molli-3 were detected in many sediment samples from sites without *Callogorgia* (Fig. S2a, Supplementary File 1: Tab A). On the first sequencing run, Molli-1 was detected in every sediment sample (mean 0.65% ± 0.19 sd, mean 19 reads ± 5.0, 24 from sites with *Callogorgia,* 14 from sites without) whereas it was detected in only 20 of 45 samples from the second sequencing run and was less abundant when present (mean 0.12% ± 0.16, mean 2.7 reads ± 1.4, present in 10/19 with *Callogorgia,* 10/26 without). Molli-3 was only detected in sediment samples from the first sequencing run (13 of 38) where up to 3 reads were detected.

Sediment from sites with *Callogorgia* did not contain a higher abundance of Molli-1 sequences on either run (Fig. S2b). All four mesophotic sediment samples were sequenced on the second sequencing run and two contained Molli-1 sequences (2 and 4 reads). Molli-1 was detected in three out of four sequencing blanks (≤5 reads, Fig. S2c).

No novel mollicute ASV was detected in any water sample. All water samples were sequenced on a third run that contained no coral samples nor any sediment.

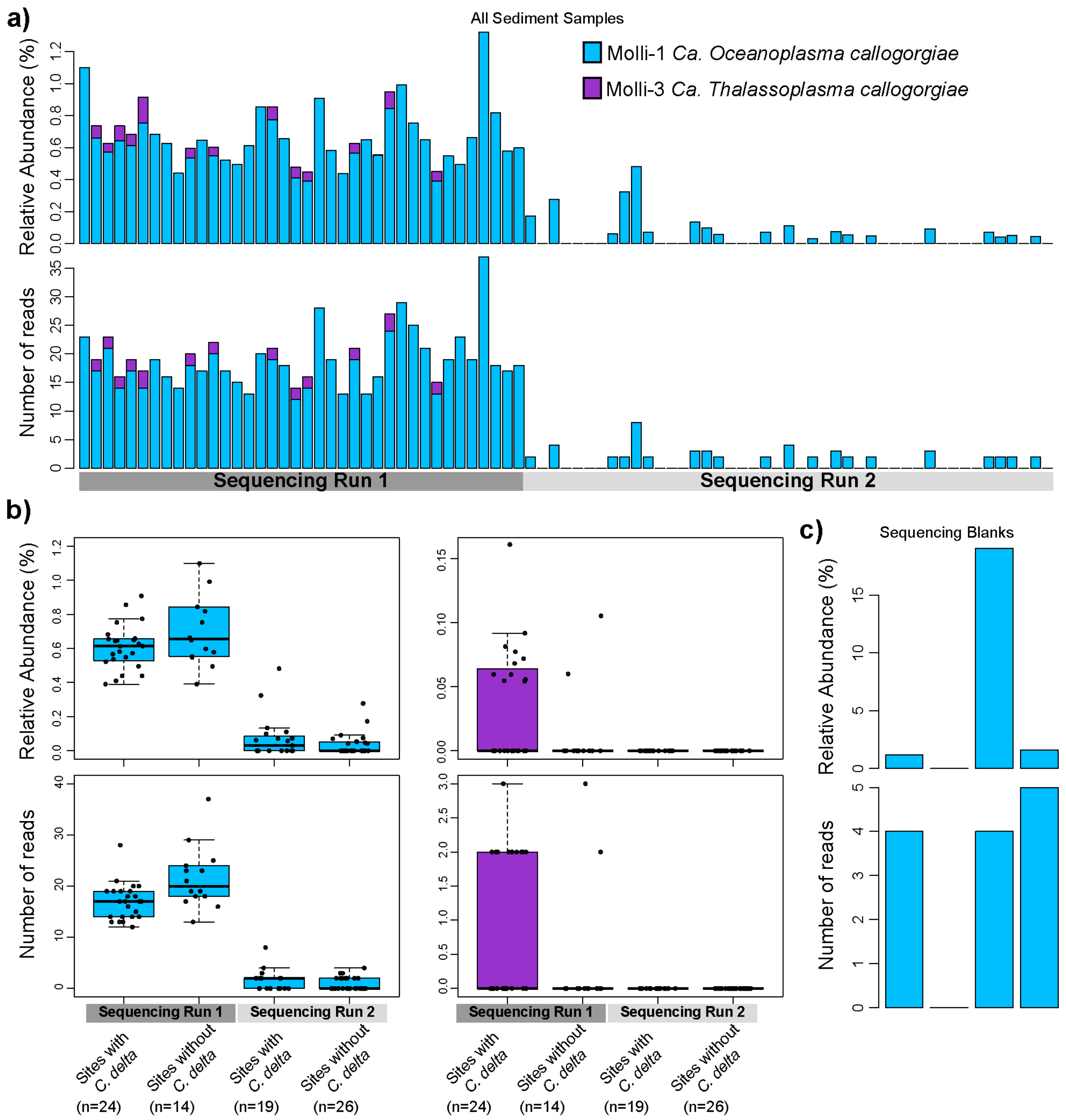

Fig. S2: Oceanoplasmataceae in sediment samples and blanks

a) Barplots showing the presence of Oceanoplasmataceae ASVs across all sediment samples ordered by increasing depth within sequencing run. Both the relative abundance and total number of reads are shown. b) Comparisons of the abundance of *Ca. O. callogorgiae* (left) and *Ca. T. callogorgiae* (right) between sediment samples from sites with *C. delta* and sites without *C. delta* showing no detectable difference. Boxes of boxplots display the 1st quartile, median, and 3rd quartile while whiskers display the minimum and maximum. All points were additionally superimposed over boxes with randomized jittering along the x-axis. c) Abundance of *Ca. O. callogorgiae* in four sequencing blanks.

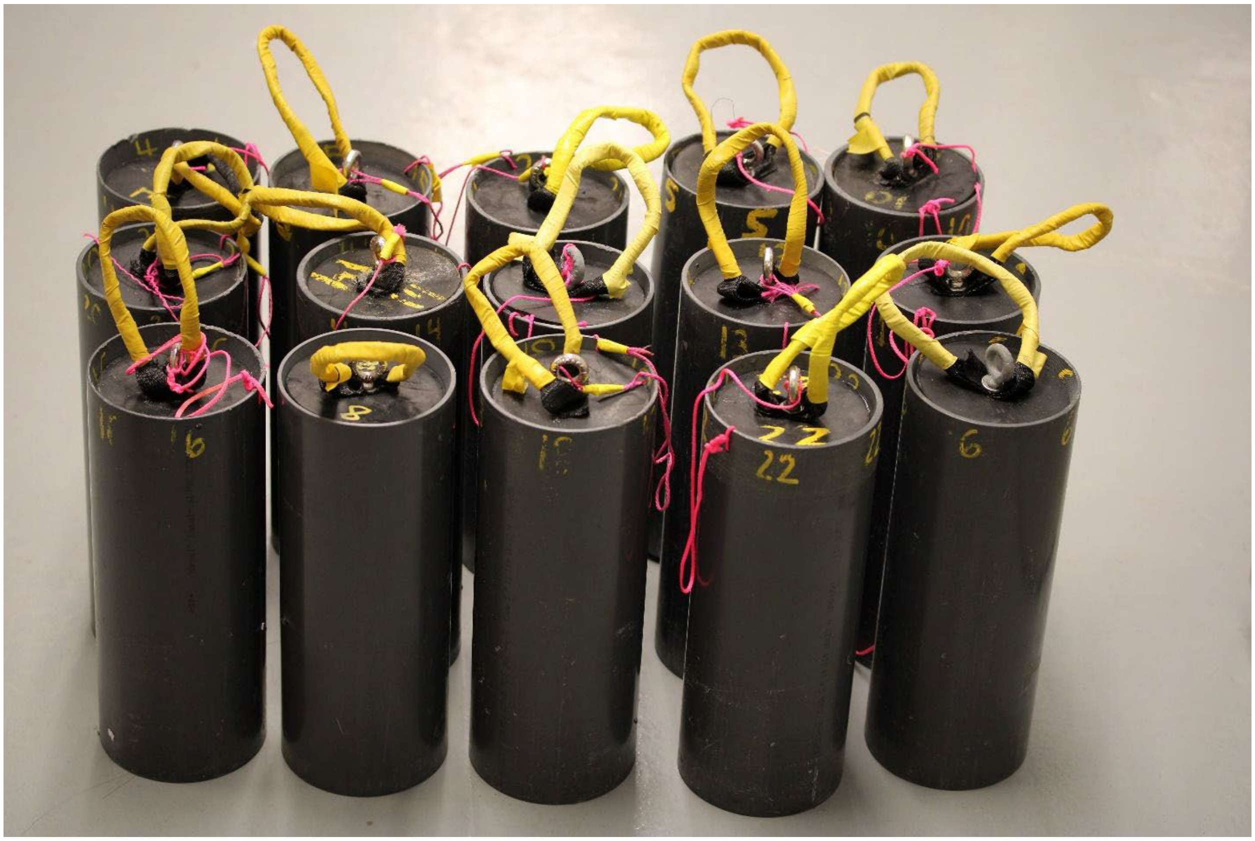

Fig. S3: Image of coral quivers

Coral quivers and bioboxes were used to collect corals in situ until ROV recovery. Details about the bioboxes and coral cutters used can be found at the following links.

ROV Hercules (Ocean Exploration Trust):

Biobox: https://nautiluslive.org/tech/rov-hercules

Coral cutters: https://sketchfab.com/3d-models/coral-cutters-728afa87f62a4693aa4ec38e0e537d19

ROV Global Explorer (Oceaneering):

Biobox: https://oceanexplorer.noaa.gov/explorations/15biolum/background/global-explorer/global-explorer.html

https://oceanexplorer.noaa.gov/explorations/15biolum/background/global-explorer/media/biobox.html

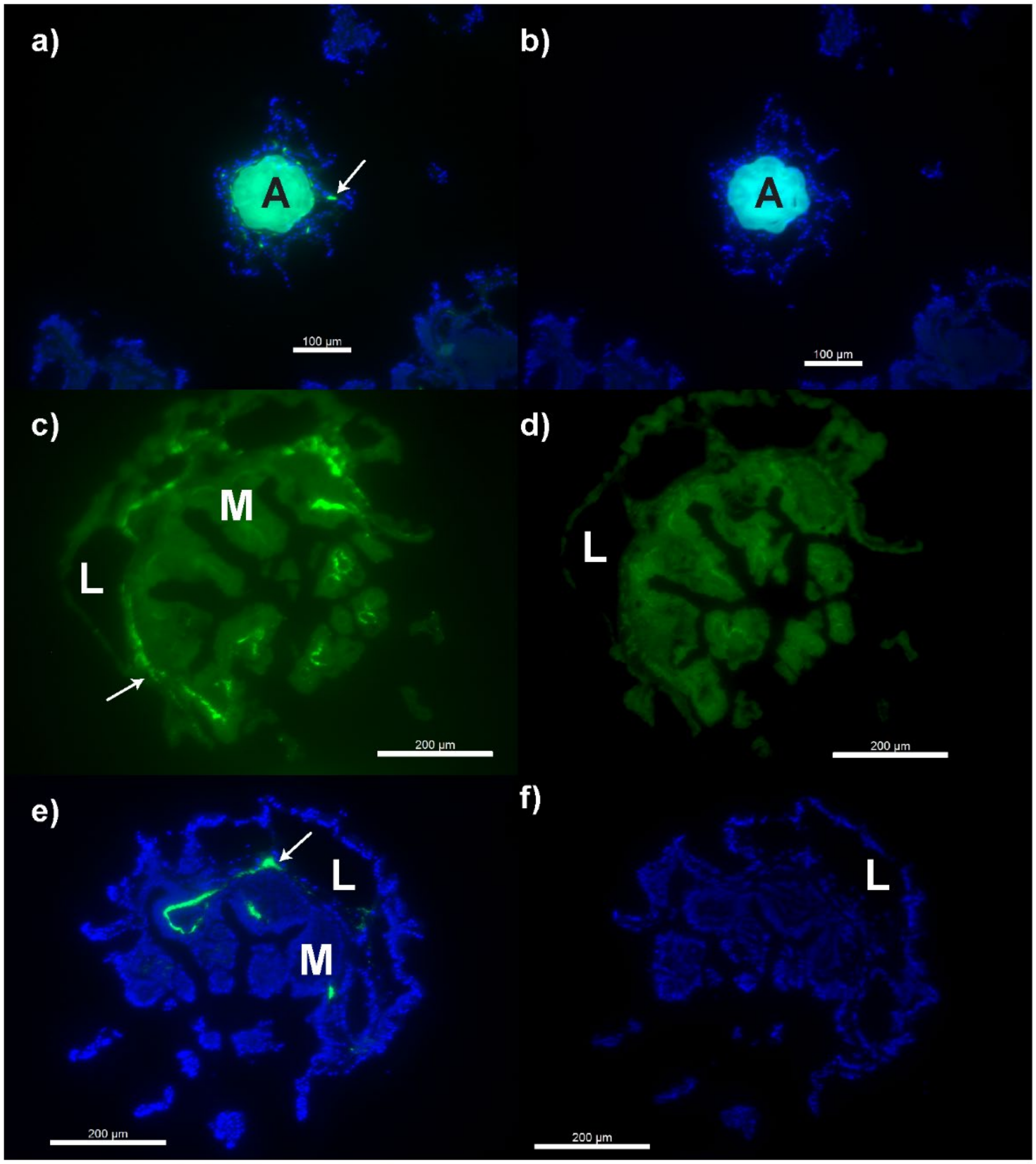

Fig. S4: Comparison of adjacent tissue sections of *Callogorgia delta*

Tissue sections were hybridized using CARD-FISH with the general eubacterial probe mix (EUB338 I-III) and DNA was stained with DAPI. Fluorescence in the emission range of DAPI is shown in blue while green shows emission in the range of the fluorophore ALEXA 488 used by both EUB338 I-III and control probes. Paired images compare adjacent sections where sections on the left were hybridized with EUB338 I-III and show bacterial signals (a,c,e) while sections on the right were hybridized with a negative control probe and only show autofluorescence in green (b,d,f). Signals were observed in transverse sections through the central axis (a,b) and transverse sections through a single polyp (c,d,e,f,). Arrows in (a) and (c) show examples of EUB338 I-III fluorescence. This pattern was observed in 4 of 5 colonies. A = central axis, G = gastrovascular cavity, M = gastric mesentery, L = sclerite lacuna. Scale bars = 100 µm (a,b,g), 200 µm (c,d,e,)

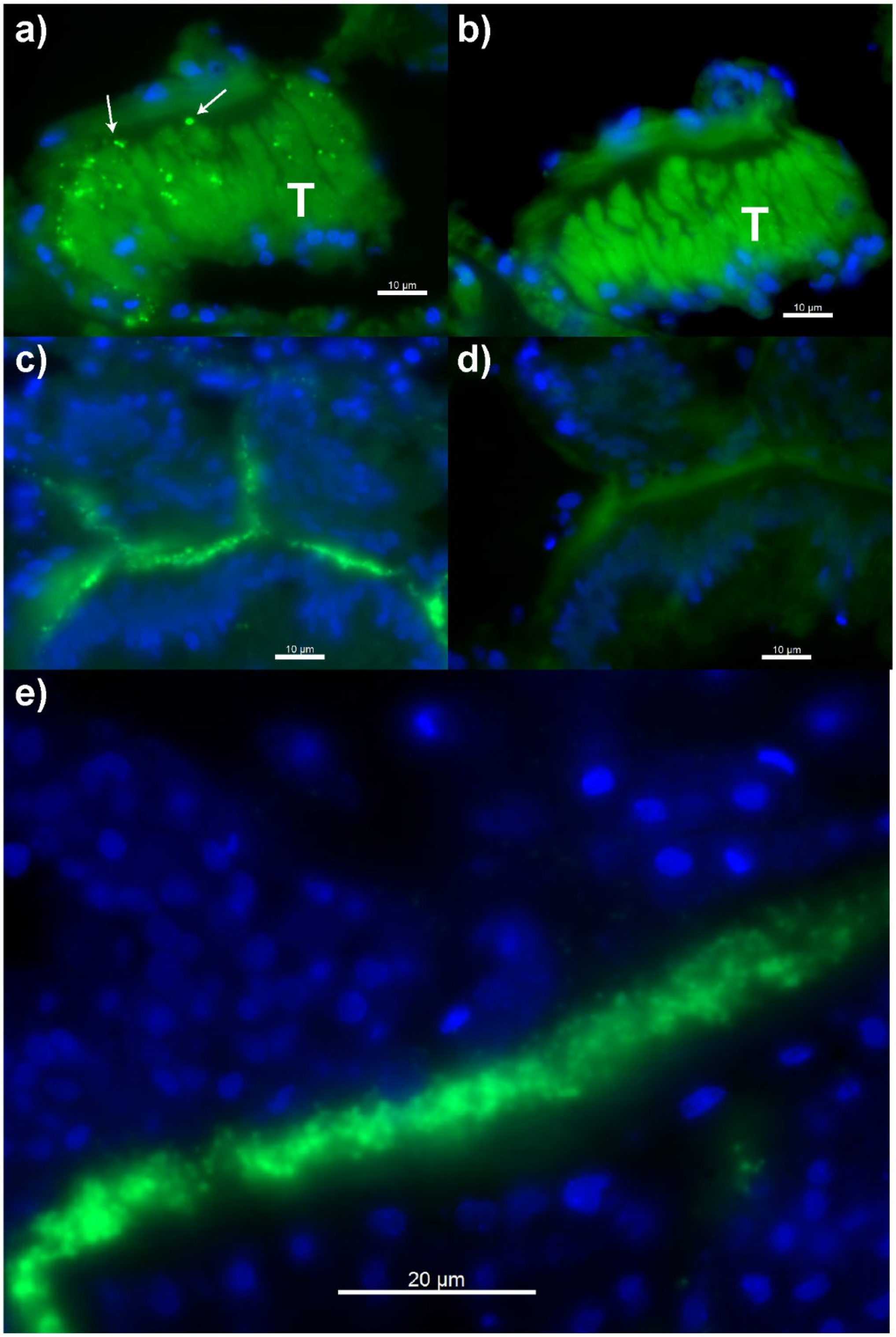
a)
T
10 µm
b)
T
10 µm
c)
10 µm
d)
10 µm
e)
20 µm

Fig. S5: Adjacent section comparisons at higher magnification

Tissue sections were hybridized using CARD-FISH with the general eubacterial probe mix (EUB338 I-III) and DNA was stained with DAPI. Fluorescence in the emission range of DAPI is shown in blue while green shows emission in the range of the fluorophore ALEXA 488 used by both EUB338 I-III and control probes. Paired images compare adjacent sections where sections on the left were hybridized with EUB338 I-III and show bacterial signals (a,c,e) while sections on the right were hybridized with a negative control probe and only show autofluorescence (b,d). Transverse sections through a polyp (a,b) show EUB338 I-III fluorescence (a, arrows) in the base of a tentacle seen in only 1 of 5 colonies. Transverse sections through the body wall of a polyp show granular (<1µm) EUB338 I-III fluorescence in the mesoglea (c-e) seen in 4 of 5 colonies. T = tentacle. Scale bars = 10 µm (a-d), 20 µm (e)
