# Supplementary material for: Discovery of deep-sea coral symbionts from a novel clade of marine bacteria with severely reduced genomes
Source: Nat Commun. 2024 Nov 4;15:9508. doi: 10.1038/s41467-024-53855-5 (PMC11535214; doi:10.1038/s41467-024-53855-5)
Supplement: Supplementary file 2 — Description of Additional Supplementary Files [file 41467_2024_53855_MOESM2_ESM.pdf]

Description of Additional Supplementary Files

File name: Supplementary Data 1

Description: Excel workbook with metadata and raw data. Tab A provides all metadata for all samples used in the 16S survey including site, species, colony names, extraction procedure, preservation method, sequencing run, depth, sampling year, whether the sample was included in figure 1 as well as the percentages and reads of ASVs Molli-1, Molli-2, and Molli-3. Tab B provides the latitude and longitude for all samples where they were available. Tab C provides the distances in meters between all samples with coordinates. Tab D provides the results from the IMNGS analysis. Tab E provides the accession numbers for all 16S sequences used in phylogenetic analyses. Tab F provides the accession numbers for all genomes used in phylogenomics analyses. Tab G provides all genomic information for *Ca. Oceanoplasma callogorgiae* including annotations, COG IDs, gene expression levels, gene positions, strand, and sequences. Tab H provides the same genomic information for *Ca. Thalassoplasma callogorgiae*. Tab I provides all the DRAM annotations for all genomes used in phylogenomics and comparative genomics analyses. Tab J provides the COG IDs for all genes in the comparative genomics analyses based on OrthoMCL. Tab K provides the OrthoANI values of comparisons of select genomes.
