# Supplementary material for: Discovery of deep-sea coral symbionts from a novel clade of marine bacteria with severely reduced genomes
Source: Nat Commun. 2024 Nov 4;15:9508. doi: 10.1038/s41467-024-53855-5 (PMC11535214; doi:10.1038/s41467-024-53855-5)
Supplement: Supplementary file 4 — Reporting Summary [file 41467_2024_53855_MOESM4_ESM.pdf]

nature portfolio

Corresponding author(s): Iliana B. Baums

Last updated by author(s): Aug 27, 2024

# Reporting Summary

Nature Portfolio wishes to improve the reproducibility of the work that we publish. This form provides structure for consistency and transparency in reporting. For further information on Nature Portfolio policies, see our Editorial Policies and the Editorial Policy Checklist.

## Statistics

For all statistical analyses, confirm that the following items are present in the figure legend, table legend, main text, or Methods section.

| n/a | Confirmed | |
|---|---|---|
| ☐ | ☒ | The exact sample size (*n*) for each experimental group/condition, given as a discrete number and unit of measurement |
| ☐ | ☒ | A statement on whether measurements were taken from distinct samples or whether the same sample was measured repeatedly |
| ☒ | ☐ | The statistical test(s) used AND whether they are one- or two-sided<br>*Only common tests should be described solely by name; describe more complex techniques in the Methods section.* |
| ☒ | ☐ | A description of all covariates tested |
| ☒ | ☐ | A description of any assumptions or corrections, such as tests of normality and adjustment for multiple comparisons |
| ☐ | ☒ | A full description of the statistical parameters including central tendency (e.g. means) or other basic estimates (e.g. regression coefficient) AND variation (e.g. standard deviation) or associated estimates of uncertainty (e.g. confidence intervals) |
| ☒ | ☐ | For null hypothesis testing, the test statistic (e.g. *F*, *t*, *r*) with confidence intervals, effect sizes, degrees of freedom and *P* value noted<br>*Give P values as exact values whenever suitable.* |
| ☒ | ☐ | For Bayesian analysis, information on the choice of priors and Markov chain Monte Carlo settings |
| ☒ | ☐ | For hierarchical and complex designs, identification of the appropriate level for tests and full reporting of outcomes |
| ☒ | ☐ | Estimates of effect sizes (e.g. Cohen's *d*, Pearson's *r*), indicating how they were calculated |

*Our web collection on statistics for biologists contains articles on many of the points above.*

## Software and code

Policy information about availability of computer code

| | |
|---|---|
| Data collection | No custom scripts were utilized to collect data. |
| Data analysis | No custom scripts were developed for this manuscript that are central to the main findings. Code used in this manuscript followed manual guidelines with extensive details described in the methods. The publicly available "Moving Pictures" Qiime2 tutorial was followed to process 16S metabarcoding data to generate an ASV table. Scripts are available upon request from the corresponding author. |

For manuscripts utilizing custom algorithms or software that are central to the research but not yet described in published literature, software must be made available to editors and reviewers. We strongly encourage code deposition in a community repository (e.g. GitHub). See the Nature Portfolio guidelines for submitting code & software for further information.

## Data

Policy information about availability of data

All manuscripts must include a data availability statement. This statement should provide the following information, where applicable:

- Accession codes, unique identifiers, or web links for publicly available datasets
- A description of any restrictions on data availability
- For clinical datasets or third party data, please ensure that the statement adheres to our policy

Raw sequence data generated or used in this study are available on the NCBI Sequence Read Archive under BioProjects PRJNA574146 (BioSample IDs SAMN12856800 – SAMN12856807) [https://www.ncbi.nlm.nih.gov/bioproject/?term=PRJNA574146] and PRJNA565265 (SRA accession numbers SRR10174410 and

SRR10174411) [https://www.ncbi.nlm.nih.gov/bioproject/?term= PRJNA565265]. Assembled 16S rRNA sequences and genomes are available under accession numbers OR679038 – 62 [Ex: https://www.ncbi.nlm.nih.gov/nuccore/OR679038.1/], CP125803 [https://www.ncbi.nlm.nih.gov/nuccore/CP125803], and JARVCM000000000 [https://www.ncbi.nlm.nih.gov/nuccore/JARVCM000000000.1/]. The Rraw microscopy images generated in this study have been depositedare available on Figshare under the following doi's: FISH, http://doi.org/10.6084/m9.figshare.26800249; thick section images, http://doi.org/10.6084/10.6084/m9.figshare.26801371; and TEM, http://doi.org/10.6084/10.6084/m9.figshare.26800081.upon request. Data used to construct figures are provided in Supplementary DataFile 1. The ASV classifications and ASV count table generated in this study was deposited on Figshare and can be accessed under the following doi: http://doi.org/10.6084/m9.figshare.26837077.

## Research involving human participants, their data, or biological material

Policy information about studies with human participants or human data. See also policy information about sex, gender (identity/presentation), and sexual orientation and race, ethnicity and racism.

| | |
|---|---|
| Reporting on sex and gender | NA |
| Reporting on race, ethnicity, or other socially relevant groupings | NA |
| Population characteristics | NA |
| Recruitment | NA |
| Ethics oversight | NA |

Note that full information on the approval of the study protocol must also be provided in the manuscript.

# Field-specific reporting

Please select the one below that is the best fit for your research. If you are not sure, read the appropriate sections before making your selection.

☐ Life sciences ☐ Behavioural & social sciences ☒ Ecological, evolutionary & environmental sciences

For a reference copy of the document with all sections, see nature.com/documents/nr-reporting-summary-flat.pdf

# Ecological, evolutionary & environmental sciences study design

All studies must disclose on these points even when the disclosure is negative.

| | |
|---|---|
| Study description | Callogorgia delta colonies that were sampled across 6 sites and 3 years were screened for the presence of novel mollicutes. Additionally, we screened two Callogorgia americana samples collected from two sites each in 2010 as well as sediment and water samples . A subset of the Callogorgia delta samples were chosen to sequence metagenomes and metatranscriptomes and for microscopy. |
| Research sample | Several groups of Callogorgia delta colonies were collected in situ from 6 locations in the Gulf of Mexico. No manipulations were performed. These sites were chosen because they are the only known locations of Callogorgia delta populations in the northern Gulf of Mexico. |
| Sampling strategy | We aimed for a minimum of 12 colonies each from three sites (MC751, GC234, and MC885). We achieved these samples plus additional samples from those sites and three other sites. All samples that were collected were processed for 16S sequencing. Sample sizes are uneven between sites and between years due to limitations of oceanographic research conducted on ships. Some dives were cut short or canceled due to weather and other events. Only three C. delta samples were collected from VK826 while all other sites had a minimum of 10. We also included four colonies of Callogorgia americana that were available from other expeditions. This was sufficient to detect whether corals from these sites hosted abundant novel mollicutes. We did not test whether the relative abundance of novel mollicutes differed across sites, years, or species because it was beyond the purpose of the study which was to determine the temporal and spatial range for the association. A subset of samples were sequenced for metagenomics. These were chosen based on the quality of DNA after extraction. The samples used to assemble mollicute genomes were sequenced for transcriptomics. For phylogenetic analyses, publicly available sequences from across the class Mollicutes were chosen to include newly sequenced marine mollicutes and members of each major family following Gupta et al. 2018 and 2019. For genomic analyses, genomes from at least 4 members of each major family were included. For microscopy, colonies were chosen based on whether we obtained metagenomes for them. |
| Data collection | Data collected  in the field included latitude, longitude, and depth which were obtained through navigation equipment and measured using a CTD on the remotely operated vehicle. These were recorded using an automated recording system or by hand by several scientists including authors SAV, IBB, and CRF. |
| Timing and spatial scale | Callogorgia delta colonies were collected from six sites spanning over 350 km in the northern Gulf of Mexico in April-May 2015, Sept-Oct 2016, and June 2017. These time frames were chosen based on when we could charter vessels in the Gulf of Mexico and when the sea state in the Gulf is conducive to ROV operations (not winter). |

| | |
|---|---|
| Data exclusions | Since replicates of some colonies were sequenced multiple times, some samples were excluded from the barplots in figure 1. The first replicate from frozen tissue and extracted with a DNeasy powersoil kit was used for each colony with replicates. |
| Reproducibility | We did not conduct any experiments. Raw data and detailed methods were provided to reproduce findings. |
| Randomization | Randomization was not utilized. In phylogenetic analyses, representative genomes across the class Mollicutes were chosen to include newly sequenced marine mollicute genomes and members of each major family following Gupta et al. 2018 and 2019. |
| Blinding | Blinding was not relevant to this study. We did not compare sample groups or have a priori hypotheses that could have benefited from blinding. |

Did the study involve field work? ☒ Yes ☐ No

## Field work, collection and transport

| | |
|---|---|
| Field conditions | Samples were collected from deep-sea habitats consisting of rocky outcrops of authigenic carbonate surrounded by soft sediment and signs of sulfide seepage. At GC249, corals had settled on the shells of vesicomyid clams that rested on the soft sediment and encircled a bed of Bathymodiolus childressi mussels that contained crude oil. Temperatures ranged from 6 to 12 degrees Celsius between sites. Samples were collected in April-May 2015, Sept-Oct 2016, and June 2017 |
| Location | Callogorgia delta colonies were collected from six sites spanning over 350 km in the northern Gulf of Mexico in 2015, 2016, and 2017. These include: Mississippi Canyon (MC) 751 (n=19 colonies, 435-443 m, 28.193 N -89.800 W) Viosca Knoll (VK) 826 (n=3 colonies, 547-559 m, 29.159 N -88.010 W) Green Canyon (GC) 234 (n=37 colonies, 505-536 m, 27.746 N -91.122 W) MC885 (n=25 colonies, 624-642 m, 28.064 N -89.718 W) GC249 (n=14 colonies, 789-795 m, 27.724 N -90.514 W) GC290 (n=10 colonies, 852 m, 27.689 N -90.646 W)<br>Callogorgia americana colonies were collected in 2010 from: Garden Banks (GB) 299 (n=3 colonies, 358-359m, 27.689 N -92.218 W) VK862 (n=2 colonies, 352-357m, 29.109 N -88.387 W). |
| Access & import/export | We obtained Letters of Acknowledgement from the NATIONAL MARINE FISHERIES SERVICE, Southeast Regional Office — 263 13th Avenue South, St. Petersburg, Florida 33701-5505 and notified the Bureau of Ocean Mineral Management (Notice NG15-004) of scientific research prior to conducting sampling. The letters of Acknowledgment (LOA) recognized the activities as scientific research in accordance with the definitions and guidance at 50 CFR 600.10. As such, the proposed activities are not subject to fishing regulations at 50 CFR 622 developed in accordance with the Magnuson-Stevens Fishery Conservation and Management Act. |
| Disturbance | Potential disturbance includes knocking down corals with the ROV and removing whole colonies. We used coral cutters to only remove a small part of coral colonies and landed the ROV in positions free of corals. |

# Reporting for specific materials, systems and methods

We require information from authors about some types of materials, experimental systems and methods used in many studies. Here, indicate whether each material, system or method listed is relevant to your study. If you are not sure if a list item applies to your research, read the appropriate section before selecting a response.

### Materials & experimental systems

| n/a | Involved in the study | |
|---|---|---|
| ☒ | ☐ | Antibodies |
| ☒ | ☐ | Eukaryotic cell lines |
| ☒ | ☐ | Palaeontology and archaeology |
| ☐ | ☒ | Animals and other organisms |
| ☒ | ☐ | Clinical data |
| ☒ | ☐ | Dual use research of concern |
| ☒ | ☐ | Plants |

### Methods

| n/a | Involved in the study | |
|---|---|---|
| ☒ | ☐ | ChIP-seq |
| ☒ | ☐ | Flow cytometry |
| ☒ | ☐ | MRI-based neuroimaging |

## Animals and other research organisms

Policy information about studies involving animals; ARRIVE guidelines recommended for reporting animal research, and Sex and Gender in Research

| | |
|---|---|
| Laboratory animals | No animals were kept in the lab. |
| Wild animals | Branches of Callogorgia delta and Callogorgia americana were collected in situ using specially designed coral cutters on the manipulator arm of a remotely operated vehicle that consisted of a cutting edge. Coral branches were removed and placed in temperature-insulated containers on the ROV until the end of the dive. Upon ROV recovery, coral samples were held in cold water until processing. Subsamples were preserved by flash freezing, in ethanol, or in fixative for microscopy. All tissue was preserved. |

| | |
|---|---|
| Reporting on sex | Sex information was not collected in this study. |
| Field-collected samples | The study did not conduct experiments on animals. The animals were preserved immediately. |
| Ethics oversight | Research on invertebrates is not subject to ethical approval. We did however follow guidelines for collecting field samples. We obtained Letters of Acknowledgement from the NATIONAL MARINE FISHERIES SERVICE, Southeast Regional Office — 263 13th Avenue South, St. Petersburg, Florida 33701-5505 and notified the Bureau of Ocean Mineral Management (Notice NG15-004) of scientific research prior to conducting sampling.  The letters of Acknowledgment (LOA) recognized the activities as scientific research in accordance with the definitions and guidance at 50 CFR 600.10. As such, the proposed activities are not subject to fishing regulations at 50 CFR 622 developed in accordance with the Magnuson-Stevens Fishery Conservation and Management Act. |

Note that full information on the approval of the study protocol must also be provided in the manuscript.

## Plants

| | |
|---|---|
| Seed stocks | NA |
| Novel plant genotypes | NA |
| Authentication | NA |
